# Venous Thoracic Outlet Syndrome Provoked by Sleeping Posture

**DOI:** 10.7759/cureus.19198

**Published:** 2021-11-02

**Authors:** Keisuke Nakabayashi, Hiroshi Ando

**Affiliations:** 1 Heart Center, Kasukabe Chuo General Hospital, Kasukabe, JPN

**Keywords:** advanced age, dynamic angiography, dementia, sleeping posture, thoracic outlet syndrome

## Abstract

A 96-year-old female with severe dementia complained of subacute onset of right arm swelling after sleep using her right arm as a pillow. Computed tomography and ultrasonography could not detect any significant findings. Nevertheless, her unilateral edema was similar to that of a venous disorder; therefore, we performed angiography of the brachial vein with the right arm in a normal position and in an abduction position; significant stenosis was seen in the latter position. We diagnosed her with venous thoracic outlet syndrome. This case was unique from the typical cases of venous thoracic outlet syndrome because of the subacute onset of symptoms, high age at onset, and discrepancies between ultrasonography and angiography findings. Dementia is the key factor explaining these features. The diagnosis of thoracic outlet syndrome is generally difficult. Although provocative physical examination maneuvers and ultrasonography are essential, dynamic testing with provocative maneuvers allows physicians to detect venous compression, even if it is difficult to capture with static imaging tests. Once the diagnosis and its etiology were confirmed, corresponding intervention, including physical therapy, is warranted.

## Introduction

A careful interview and physical examination are the most important aspects of internal medicine. In addition, many medical examinations require the patient's cooperation, such as positioning and breathing. However, it is often difficult to conduct an adequate examination in the recent aging society, especially for patients with dementia. Here, we present a case of venous thoracic outlet syndrome in an elderly woman with an atypical course and difficulties in the examination due to her severe dementia.

## Case presentation

**Figure 1:**
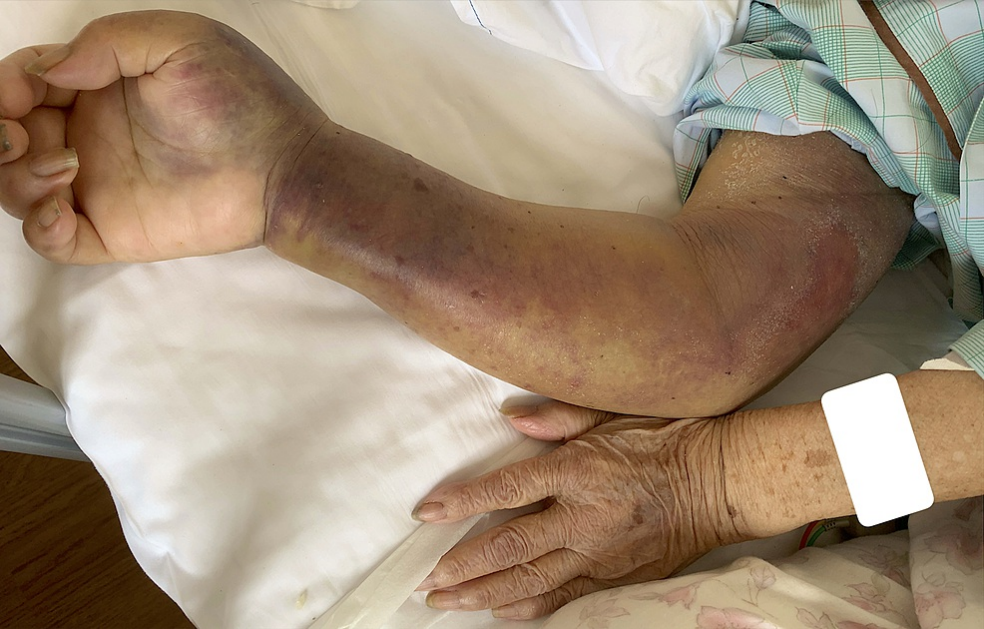
Physical examination findings of the right arm. The right arm with severe swelling, purpura, and skin tension after waking.

A 96-year-old female with hypertension and severe dementia complained of subacute onset of right arm swelling and pain after waking up. Her advanced state of dementia made it difficult to obtain a sufficient history taking and physical examination; however, care provider had confirmed that her right arm had been in a normal state last night and witnessed that she slept using her right arm as a pillow. Her right arm showed severe purpura and skin tension; however, her radial artery was palpable (Figure 1).

**Figure 2:**
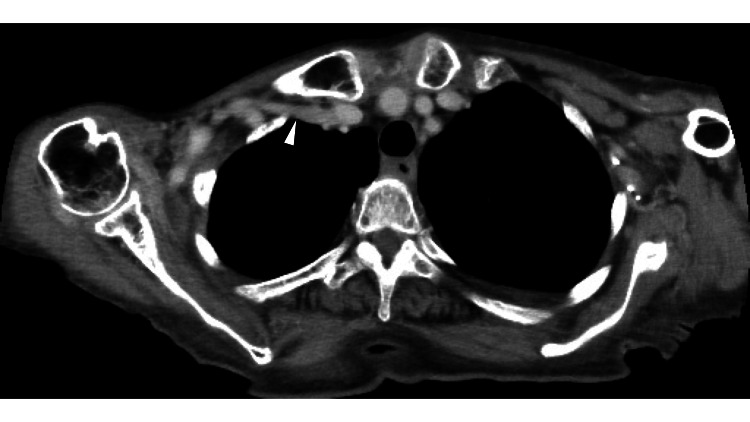
Computed tomography. Computed tomography in the normal position shows no significant stenosis of the right subclavian vein (white arrowhead).

An orthopedist referred her to our cardiovascular department. Computed tomography showed no significant stenosis of the right subclavian vein (Figure 2).

**Figure 3:**
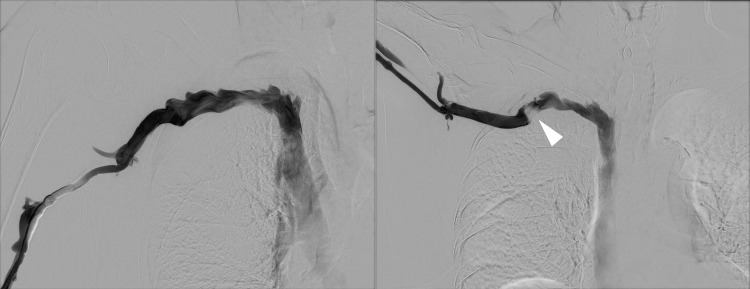
Angiography. Left panel: Angiography of the brachial vein in the normal upper limb position, which shows no significant stenosis of the right subclavian vein. Right panel: Abduction position induces significant stenosis of the right subclavian vein (white arrowhead).

Ultrasonography in both normal and abduction positions also could not detect any significant findings. In addition, computed tomography and ultrasonography did not reveal any thrombus in the proximal and distal right subclavian vein. Nevertheless, her unilateral edema was similar to that of a venous disorder; therefore, we performed angiography of the right brachial vein in a normal position (Figure 3, left panel) and in an abduction position (Figure 3, right panel).

Significant stenosis was seen in the latter position. In the absence of nervous and arterial symptoms, we diagnosed her with venous thoracic outlet syndrome. We were hesitant to pursue an invasive surgical strategy and anticoagulation due to the severity of her dementia; therefore, we used a bust-band to fix her right arm in the adduction position and guide her care provider not to use her arm as a pillow, which resolved her symptoms.

## Discussion

The thoracic outlet is an anatomical area in the lower neck defined as a group of three spaces (interscalene triangle, costoclavicular space, and subcoracoid space) between the clavicle and the first rib. Three important neurovascular structures (brachial plexus, subclavian artery, and subclavian vein) pass through this area [1]. Compression of this area causes distinct symptoms. According to the compressed structure, thoracic outlet syndrome is divided into three subgroups: neurogenic, arterial, and venous. Venous thoracic outlet syndrome has the pathognomonic presentation of the upper limb swelling, cyanosis, heaviness, and pain. In addition, venous thoracic outlet syndrome is more common in younger and able-bodied individuals due to its association with repetitive upper limb activity [2].

This case was unique from the typical cases of venous thoracic outlet syndrome. Firstly, she had subacute onset of symptoms. Usually, venous return disorders gradually demonstrate edematous change. Dementia and sleeping posture are the causes; she might have used her right arm as a pillow in the abduction position without noticing the abnormality until she woke up. Secondly, she had a relatively high age at onset. Dementia might have affected this late onset. Usually, pain or discomfort awakens cognitively normal patients. Unawareness of primary symptoms leads to a deteriorated condition by the first medical contact. Thirdly, there are discrepancies between ultrasonography and angiography findings. Longley et al. reported 92% specificity and 95% sensitivity using ultrasonography in the diagnosis of venous thoracic outlet syndrome [3]. However, her dementia symptoms prevented adequate positioning and examination time, which obscured the area of interest. Angiography can cancel this restriction. Therefore, we performed angiography, administering an intravenous sedation agent and using passive positioning, which clearly detected the etiology of this phenomenon.

The diagnosis of thoracic outlet syndrome is generally difficult. Although physical examination maneuvers and ultrasonography are essential, dynamic testing with provocative maneuvers allows physicians to detect venous compression [4], even if it is difficult to capture with static imaging tests. Once the diagnosis and its etiology were confirmed, corresponding intervention, including physical therapy, is warranted.

## Conclusions

The diagnosis of thoracic outlet syndrome is generally difficult, especially in patients with dementia, who are difficult to interview and uncooperative with testing. In such situations, a provocative examination under sedation might be useful to understand pathophysiology.
